# Factors influencing underrepresented geoscientists’ decisions to accept or decline faculty job offers

**DOI:** 10.21203/rs.3.rs-4224855/v1

**Published:** 2024-04-29

**Authors:** Margaret L. Duffy, Liza Y. Barnes, Christopher D. Wirz, Meghana Ranganathan, Mara A. Freilich, Lyssa M. Freese, Ellen Lalk, Julia Wilcots

**Affiliations:** 1 Climate and Global Dynamics Laboratory, NSF National Center for Atmospheric Research, PO Box 3000, Boulder, 80307, CO, USA.; 2 LeBow College of Business, Drexel University, 3141 Chestnut Street, Philadelphia, 19104, PA, USA.; 3 Mesoscale and Microscale Meteorology Laboratory, NSF National Center for Atmospheric Research, PO Box 3000, Boulder, 80307, CO, USA.; 4 School of Earth and Atmospheric Sciences, Georgia Institute of Technology, 311 Ferst Drive, Atlanta, 30332, GA, USA.; 5 Cooperative Program for the Advancement of Earth System Science, University Corporation for Atmospheric Research, PO Box 3000, Boulder, 80307, CO, USA.; 6 Division of Applied Mathematics, Brown University, 170 Hope Street, Providence, 02906, RI, USA.; 7 Department of Earth, Environmental, and Planetary Sciences, Brown University, 324 Book Street, Providence, 02912, RI, USA.; 8 Department of Earth, Atmospheric, and Planetary Sciences, Massachusetts Institute of Technology, 77 Massachusetts Avenue, Cambridge, 02139, MA, USA.; 9 Department of Global Ecology, Carnegie Institution for Science, Stanford, 94305, CA, USA.; 10 Department of Geosciences, Princeton University, Guyot Hall, Princeton, 08544, NJ, USA.

**Keywords:** Hiring, Diversity, Faculty, Education

## Abstract

Many geoscience departments are taking steps to recruit and retain faculty from underrepresented groups. Here we interview 19 geoscientists who identify as an underrepresented race or gender who recently declined a tenure-track faculty job offer. A range of key factors influenced their decisions to accept or decline a position including commitment to diversity, equity, and inclusion (DEI) including personal identities, DEI initiatives, and mentorship; (in)civility during job interviews; values revealed in negotiation; and compatibility with personal life including family and geography. Many of the participants experienced hiring processes inconsistent with existing recommendations to increase faculty diversity. Therefore, we leverage our results to provide actionable recommendations for improving the equity and effectiveness of faculty recruitment efforts. We find that departments may doubly benefit from improving their culture: in addition to benefiting current members of the department, it may also help with recruitment.

## 1 Introduction

There is a lack of racial, ethnic, and gender diversity amongst geoscience faculty. Only 13.6% of tenured and tenure-track geoscience faculty in the US identify as underrepresented racial and/or ethnic minorities as compared with 38% of the US population^1^ and fewer than 30% of tenured and tenure-track geoscience faculty identify as women [1–3]. Throughout the early 2000s, the under-representation of Ph.D. students and faculty of color in the geosciences has persisted [1]. Meanwhile, the fraction of graduate students and faculty who are women has increased over the past few decades [1, 3, 4]. [3]. However, Ranganathan et al., 2021 estimate that gender parity in hiring will not translate to gender parity amongst US geoscience faculty until around the year 2056 unless further interventions are made [3].

There are a number of reasons why geoscience departments^2^ are motivated to improve faculty diversity. Increasing the diversity of geoscience faculty is fundamentally important for equity within academia [5] and has implications for the impact of science on society. The demographics of geoscience departments have implications for their functioning. If diversity is managed correctly – by cultivating a sense of inclusion and belonging [6] – it can promote innovation [7]. Diverse groups perform better than homogeneous groups in difficult tasks, as they pay more attention to different perspectives [8], engage in greater information sharing, and incorporate that information more effectively into decision-making [9]. These tendencies are critical for geoscience research, which is societally relevant and can have cascading impacts beyond the academy. Greater diversity can also reduce the likelihood of turnover of employees from underrepresented groups, especially when early-career employees see representation in higher ranks of the organization [10]. Increasing representation of scientists from underrepresented groups can reduce implicit biases and stereotype threat [11]. In particular, role models for graduate students can advance their career trajectories and benefit their mental health [12]. These benefits are especially important for academic departments, tasked with training students and other early-career geoscientists.

Despite attempts to diversify the geosciences, geoscientists holding underrepresented racial, ethnic, and gender identities still face more barriers to successful participation than geoscientists from well-represented groups [13]. For example, a 2019–2020 survey revealed that geoscientists of color, women, and nonbinary geoscientists were more likely to report behavior such as discrimination, harassment, and mistreatment than other geoscientists [14]. Likely as a result, geoscientists from these underrepresented groups were more likely to avoid colleagues and skip professional events than their peers [14]. Further, a staggering half of women and nonbinary geoscientists and geoscientists from some racial minority groups have considered leaving their departments, a rate higher than that of other geoscientists [14]. Therefore, the obstacles to hiring diverse faculty are significant and need to be examined to find effective ways to surmount them [13].

Previous research has recommended a number of interventions to diversify faculty, including supporting dual-career couples; implementing family-friendly policies; improving mentorship, career development, and networking opportunities; increasing the visibility of diverse faculty; and changing hiring practices. Procedures supporting dual-career couples and family responsibilities are particularly important for the recruitment and retention of women faculty because women faculty in the natural sciences are disproportionately likely (48% of women and 35% of men) to have academic partners [15]. Successful interventions to improve hiring for dual-career couples include appointing a neutral liaison to support dual-career couples, using resources such as the higher education dual career network (HEDCN) and higher education recruitment consortium (HERC) or appointing a partner to an academic position within the university (with funding) [16]. An academic’s childbearing years often overlap with critical career stages, including graduate student, postdoctoral, and assistant professor positions. Moreover, parenthood affects women’s preferences about work-life balance more than men’s [17]. Successful interventions for supporting parents include time and space for breastfeeding [18], paid parental leave, part-time work options, funding for backup child care, and on-site child care [19].

Effective mentorship of junior faculty can increase retention [20]. Successful interventions to improve mentoring include formal mentoring programs, such as Mentoring Physical Oceanography Women to Increase Retention (MPOWIR) [20], and department-hosted career development and networking events [19–21]. Awards can help propel an early-career scientist, but selection can be subject to implicit biases without care. Successful interventions have included awards for excellence in mentorship and taking steps to reduce implicit bias in award nominations and selection [11, 19]. Furthermore, some departments have seen success by making changes to their hiring practices [19]. Examples of such practices include changes in how positions are advertised [19, 21], conducting broader searches [22], changes to the composition of hiring committees [19, 21], educating the search committee on best practices [11, 19, 21], and cluster hiring [23]. In addition to making their own practices more inclusive, search committees can evaluate candidates based on their demonstrated commitments to mentorship and diversity, equity, and inclusion (DEI).

The research described above has primarily studied outcomes associated with various interventions. That is, researchers implement an intervention and then examine how their department or institution’s demographics change. However, to our knowledge, there is no research about how geoscientists holding underrepresented racial, ethnic, and gender identities perceive these interventions in practice, and there is an overall lack of narratives about their job search experiences. Therefore, we evaluate the faculty-job search experiences of geoscientists from these underrepresented groups. We interview 19 geoscientists who have recently declined at least one tenure-track faculty job (see Methods) about the factors that influenced their decision to decline (an) offer(s) and/or accept a different offer. These interviews highlight the key factors that influenced their decisions: commitment to DEI including personal identities, DEI initiatives, and mentorship; (in)civility during job interviews; values revealed in negotiation; and compatibility with personal life including family and geographic preferences.

## 2 Findings

**Fig. 1:**
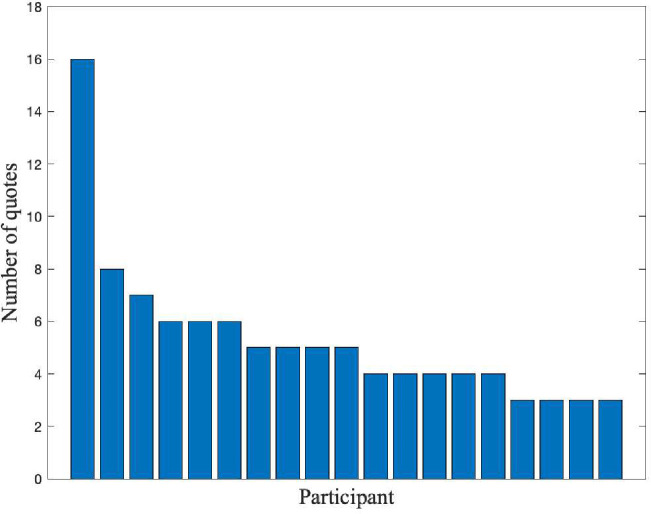
Number of quotes per participant.

The data presented in this paper include exemplary quotes from the participants. The quotes are organized into tables by theme and each quote has a Quote ID (e.g. Identities 1). In the text, we summarize the range of responses for each theme, referring to the quotes in the tables. For example, to refer to the quote in Table 1 with Quote ID Identities 1, we write “(Table 1: Identities 1).” There are 101 quotes in total, and the distribution of quotes across participants is shown in Figure 1. Quotes that pertain to a specific job include whether the participant accepted, declined, or did not receive an offer for that job. There are at least 3 quotes per participant, and 3 to 8 quotes are used for all but one participant, who gave 16 quotes. This participant had more job interviews than most participants, which they told us about in detail. Each of the 19 participants gave unique reasons for declining and accepting offers. However, a few common themes emerged, which we determine by evaluating each participants’ strongest factor(s), as described in Section 4.2. We further explore the themes that participants discussed during their interviews which relate to personal identities and personal lives. Those themes are: commitment to DEI including personal identities, DEI initiatives, and mentorship; (in)civility during job interviews; values revealed in negotiation; and compatibility with personal life including family and geographic preferences. We also include a few miscellaneous quotes on other themes.

### 2.1 Departmental commitment to diversity, equity, and inclusion

Similar to other aspects of department culture (see Section 2.2), participants gained a strong impression of a department’s commitment to DEI during the hiring process, and many participants considered this commitment in their decisions. Departments reveal a commitment to DEI through support for DEI initiatives, discussing DEI with candidates during the search process, respecting candidates’ personal identities during the search process, demographics of the department, experiences of department members from underrepresented groups, mentorship for junior faculty, and valuing mentorship of students.

#### 2.1.1 Personal identities

Participants’ personal identities were integral to their job searches. Many participants were looking for a department, university, and/or municipality in which their personal identities were represented (Table 1: Identities 1–2). Participants often mentioned their personal identities in describing their geographic preferences (Table 1: Identities 3). Identities also played a role in how participants viewed their job interview and negotiation experiences (Table 1: Identities 4). As we will discuss in Sections 2.2 and 2.3, some participants’ experiences during the interview and negotiation processes were a direct reflection of their personal identities (Table 4: Interviews 1, Interviews 10–11, Interviews 17; Table 5: Negotiation 2–5). Several participants also mentioned feeling tokenized^3^ during the hiring process (Table 1: Identities 5–7). For example, participants felt tokenized if they felt a department or institution was only hiring them to improve their diversity statistics. Several participants mentioned the importance of role models (Table 1: Identities 8), and some specifically mentioned wanting role models who share their views about being a member of an underrepresented group.

While our only demographic criteria for selecting participants was race, ethnicity, and gender, participants mentioned several other identities which influenced their decisions (Table 1: Identities 9–11). Country of origin (Table 1: Identities 9), sexual orientation (Table 1: Identities 10), and status as a first-generation college student (Table 1: Identities 11) were all mentioned by participants, indicating that the combination of a candidate’s various identities influences their decision making.

#### 2.1.2 DEI initiatives

Participants offered a range of experiences with respect to diversity, equity, and inclusion (DEI) during the hiring process. Several participants said they were looking for a department with a commitment to DEI (Table 2: DEI 1–2; Table 10: Strongest 8) or, similarly, were deterred by departments that did not show a commitment to DEI (Table 2: DEI 3–5), and many participants were able to detect a department’s commitment to DEI during the hiring process (Table 2: DEI 2–5).

During hiring process, some participants enjoyed positive experiences with respect to DEI (Table 2: DEI 2; Table 10: Strongest 8). However, several participants noticed that DEI came up more with students and other junior people in the departments than with senior faculty (Table 2: DEI 3), which one participant described as “odd.” Several participants who are very committed to DEI work wondered if it might not be a coincidence that they were not offered jobs in departments that did not appear to value DEI (Table 2: DEI 4). Several participants were deterred by a perceived lack of commitment to DEI, including several participants who questioned whether members of the search committee read what they wrote about DEI (Table 2: DEI 5). During an on-campus interview, a professor insinuated to the the participant that they were lying about their DEI work (Table 4: Interviews 12). Overall, many participants were impressed by departments with a strong commitment to DEI and/or deterred by departments which demonstrated a lack of commitment to DEI.

#### 2.1.3 Mentorship

Mentorship can help geoscientists from underrepresented groups navigate the barriers associated with their personal identities. Several participants emphasized the importance of mentorship from their Ph.D. and postdoctoral advisors (Table 3: Mentorship 1–2), peers (Table 3: Mentorship 3), and mentorship in teaching (Table 3: Mentorship 4). One participant even stated that they felt mentorship outweighed compensation in their job search (Table 3: Mentorship 5). Some participants did not feel that they had received adequate mentorship by the time they were applying for faculty jobs (Table 3: Mentorship 6). Relatedly, for many participants, a job with mentorship duties appealed to them (Table 3: Mentorship 7–8).

### 2.2 (In)civility during job interviews

Participants reported a range of experiences during their job interviews, some of which improved their perception of the job and some of which worsened their perception of the job. Things that participants were impressed by during their job interviews included: departments considering their needs (Table 4: Interviews 1), positive interactions with faculty (Table 4: Interviews 2–3), a good sense of camaraderie amongst the faculty (Table 4: Interviews 4–5), and meaningful interactions with students (Table 4: Interviews 6–8).

A dismayingly large number of participants reported specific job interview experiences that were very negative. Two participants had unsettling interactions with respect to professors in the department who had previously been publicly disciplined for their behavior (Table 4: Interviews 9). Additionally, two participants were asked illegal questions (Table 4: Interviews 10; Table 10: Strongest 1). Several participants reported hearing disparaging comments during a job interview (Table 4: Interviews 11–13). Multiple participants perceived a lack of interest from the faculty during their job interview (Table 4: Interviews 14–16; Table 10: Strongest 3) with behavior ranging from not having read the statements in their applications to faculty missing their scheduled meeting with a participant. One participant noted that there is unwelcome pressure to drink during job interviews (Table 4: Interviews 17). Importantly, several of these experiences were directly related to the participant’s personal identities (Table 4: Interviews 1, Interviews 10–11, Interviews 17).

Overall, we find that participants got a strong impression of the department’s culture during on-campus visits, including underlying issues, and that this impression was often a factor in decision-making. Several participants also perceived that the way they confronted issues during their job interviews affected whether or not they got an offer, which may be one way that institutions maintain barriers facing geoscientists from underrepresented groups.

### 2.3 Values revealed in negotiation

Beyond establishing the material support that a participant would have if they were to accept the job offer, negotiations revealed to participants how supportive the institution would be of them as employees. Several participants had confusing negotiation experiences, particularly being asked what they wanted rather than being made an offer first (Table 5: Negotiation 1) and being unsure when to mention their family needs (Table 5: Negotiation 2–3). Family often came up for many participants with respect to partner hires: for many participants an opportunity for a partner was a strong consideration (Table 5: Negotiation 4–5) and often among the strongest considerations (Table 10: Strongest 5, Strongest 9, Strongest 15, Strongest 17). Several more participants were disheartened by the negotiation process, including being lowballed (Table 5: Negotiation 6), being told “we’re fine if you don’t come here” (Table 5: Negotiation 7), being told that an offer might need to be rescinded in response to asking for a course release (Table 5: Negotiation 8), and disparaging comments during a negotiation about lab space (Table 5: Negotiation 9). The offer itself was a strong factor for many other participants (Table 5: Negotiation 11–13; Table 10: Strongest 6–7). The timing of the offer, particularly the timeline to respond to the offer, was a factor for several participants (Table 5: Negotiation 14–15; Table 10: Strongest 4) Identity can factor into negotiation tactics and the strength of negotiation position. Several participants explicitly mentioned ways that identity was realized through negotiation, including a lack of support (Table 5: Negotiation 2–5) or a perception that they were being made an unreasonable offer because of their identity (Table 5: Negotiation 6). Several participants mentioned that the offer and negotiation process signaled whether or not they would feel valued (Table 5: Negotiation 7, Negotiation 9, Negotiation 11). The offer was such a strong factor for so many participants across different personal identities, that we believe it warrants more discussion.

For most participants, salary was the most important part of the offer, but for some, it was lab space. For seven participants, the offer was inadequate and they ultimately declined the offer, and two accepted despite poor offers. A surprising number of participants mentioned very low salary offers (Table 5: Negotiation 10). In fact, five participants described salary offers that were lower than their postdoctoral positions’ salaries (Table 5: Negotiation 12; Table 10: Strongest 7). Low salary offers were a deterrent when participants felt the offers were not enough to support themselves and their families and for some participants it raised concerns about how faculty are treated (Table 5: Negotiation 11). Many participants were looking for enough compensation to buy or rent a home large enough for their family, to be able to afford childcare, to have enough money to travel to see family, and/or to be able to support a partner if a partner hire was not an option. It is important to note that socioeconomics and race intersect in the US [24]. Further, several participants were looking for lab space commensurate with their research goals. Additionally, several participants described wanting course releases in the early part of their faculty job in order to have time to prepare their course materials while building their research groups. Over half of the participants described offers that they felt were lacking in one or more of these areas. Three additional participants mentioned retention offers, two accepted (Table 10: Strongest 9) and one declined.

Overall, we find that various aspects of the negotiation process, from the process to the offer itself, influenced participants’ decisions, and often negatively.

### 2.4 Compatibility with personal life

Every participant mentioned personal life considerations. They all mentioned family, regardless of relationship status or parental status. As one participant noted, there are challenges associated with moving for a job whether single or in a relationship (Table 6: Family 1). Relatedly, geographic preferences were common among participants’ strongest reasons for accepting or declining an offer (Table 10: Strongest 1, Strongest 8–14, Strongest 16). For many participants, geographic preferences were tied to family, including being close to their partner or family, their partner’s family, their partner’s job, and their partner’s geographic preferences.

#### 2.4.1 Partner and Family

In general, participants with partners considered the preferences and needs of their partners in deciding whether or not to apply to a job (Table 6: Family 2), in negotiating an offer (Table 6: Family 3), and ultimately in deciding whether or not to accept an offer (Table 10: Strongest 5, Strongest 11–12, Strongest 15, Strongest 17–18). Nonetheless, several participants chose not to mention their partner during job interviews (Table 6: Family 4). Participants with children and participants who planned for children in the future considered this in their job search (Table 6: Family 5). Additionally, seven participants expressed a desire to be close to relatives (Table 6: Family 6; Table 10: Strongest 8–9, Strongest 11–12), and 10 participants considered the geographic preferences of their partner, partner’s job, or partner’s family (Table 10: Strongest 5, Strongest 9, Strongest 11–13, Strongest 15, Strongest 17–18). While it is clear that partners added a geographic constraint for many participants, one participant mentioned the unique difficulties of being single (Table 6: Family 6). Further, four participants mentioned looking for additional evidence of work-life balance in their interactions with faculty (Table 6: Family 7).

Six of the participants requested partner hires as part of the negotiation and were met with a mix of responses. Two of them successfully negotiated partner hires and accepted the position. Three were met with negative responses and ultimately declined the offers. One participant asked for a partner hire at two different institutions, one gave a negative response and the other found an opportunity for their partner but it was a less exciting opportunity that the partner’s existing position (Table 5: Negotiation 4). The participant declined them both. In addition to the six participants who requested partner hires from the institution(s) that made them a(n) offer(s), ten participants mentioned their partner playing a role in their decision. Overall, it is clear that partners and families were strong factors for most participants.

#### 2.4.2 Geographic preferences

Geographic preferences were common among participants’ strongest reasons for accepting or declining an offer (Table 10: Strongest 1–6, Strongest 8, Strongest 10, Strongest 13–14). In general, participants did not feel that they could be picky about geography, despite having preferences (Table 7: Geography 1–2). In addition to proximity to family and partner preferences, state and local politics (Table 7: Geography 3–4), feeling safe in a community (Table 7: Geography 5), race relations (Table 7: Geography 6), diversity (Table 7: Geography 7), and a preference for a city (Table 6: Family 6; Table 7: Geography 7) were the most cited reasons for having a geographic preference. Geographic presence was such a strong factor for so many participants across different personal identities that we believe it warrants more discussion.

Ten participants mentioned the politics of certain states or regions. This sentiment was consistently a negative one about moving to a state with conservative politics (e.g. Texas, Florida). The participants’ feelings about moving to a conservative state ranged from a willingness to try it to a dealbreaker. Further, six participants mentioned wanting to be in a municipality with diversity and where they would feel comfortable given their identities. This is in addition to four participants who mentioned wanting to be in a diverse department or university. Overall, 10 of the 19 participants mentioned wanting to be in a diverse community.

Focusing on the political preferences of participants, every participant who mentioned a political preference preferred liberal areas to conservative areas. Most participants who mentioned political preferences described recent changes to the political landscape in some states, such as interference with the tenure process, changes in access to reproductive care in following the overturn of Roe V. Wade in 2022, recent restrictions in access to gender-affirming care, and the illumination of racial tension in some cities (e.g. Minneapolis, MN and Louisville, KY). Participants were wary of some of these changes for the sake of themselves, their families, and their prospective students.

### 2.5 Other

There were several other insightful responses that do not fit into any of the previous categories. Existing recommendations mention broadening searches to increase the diversity of the applicant pool. However, some participants applied to only a very small number of jobs (Table 8: Other 1) while others applied to many (Table 8: Other 2), but participants generally had negative feelings about broad calls (Table 8: Other 1–2). Several participants were deterred by positions that requested reference letters up front (Table 8: Other 3). The reputation of a department’s culture, positive or negative, influenced some participants’ decisions to apply for a job (Table 8: Other 4–6). One participant succinctly summarized the experience as “very personal” (Table 8: Other 7). Another explicitly stated a concern about safety on college campuses (Table 8: Other 8). One participant described struggling with impostor syndrome after securing a competitive tenure-track position (Table 8: Other 9). And finally, participants noted pressure to accept a tenure-track job offer or a stigma against declining one because of how tenure-track jobs are perceived (Table 8: Other 10).

## 3 Discussion

Each participant’s unique hiring experiences when combined yielded a rich dataset that highlights several areas of improvement for departmental hiring practices. Several of these practices have been studied in depth in previous research and several more warrant future research. Nonetheless, given the urgency of improving faculty hiring in the geosciences, especially for geoscientists from underrepresented groups, we compile some recommendations for hiring practices based on our findings. These recommendations are described in the text below and summarized in Table 9.

Departments can improve the experience for candidates by diversifying their departments and speaking respectfully about personal identities, even ones they may not be aware of. In particular, helping candidates with any visa needs they may have [25], and using candidates’ correct pronouns can help make an offer more appealing. Participants often felt tokenized during the hiring process. Actions that led a participant to feel tokenized during the hiring process included overemphasizing how diverse a new cohort was, pressuring candidates to speak about their personal identities during the job interview, and generally making participants feel valued only for their contributions to diversity. Participants expressed a desire to feel like they would be valued for their contributions beyond their contributions to diversity and to feel like they were going to be supported by their department. Therefore, being careful not to tokenize candidates from underrepresented groups can help make a department more appealing.

Departments can improve the experience for candidates by actively engaging in and supporting DEI work. In particular, departments can be more attractive to candidates by improving the diversity of their departments and talking about DEI in a well-informed way. Because participants were wary of departments where DEI work fell predominantly on students and young faculty, encouraging senior faculty to engage in DEI work can help make an offer more appealing to candidates.

Departments and institutions can improve their hiring process by having strong mentorship systems for new hires and describing those mentorship systems to candidates. The importance of mentorship has been identified for improving gender and racial/ethnic diversity [20, 26]. Consistently, participants valued receiving good mentorship, and many participants expressed an interest in mentoring students. Therefore, prioritizing mentorship across career stages can make an offer more appealing to candidates.

Importantly, we find that candidates gain a strong impression of a department’s culture during the hiring process; underlying issues are often made visible to candidates. The kinds of problems that participants witnessed during campus visits include student dissatisfaction, faculty dissatisfaction, infighting within the faculty, conflicts surrounding faculty members who have a reputation for misconduct (such as sexual harassment), and unprofessional behavior (such as disparaging comments and shouting). Because many participants were able to get a strong sense of the department culture during their job interviews, and because many participants were looking for a job with a good culture and work-life balance, supporting improvements to departmental culture and the work-life balance of existing faculty may be helpful in recruitment. In short, departments may doubly benefit from improving their culture: in addition to benefiting current members of the department, it may also help with recruitment.

It is clear that some departments are still unaware of hiring best practices because two participants reported being asked illegal questions and several more reported disparaging comments. Departments can improve the experience for candidates by providing any necessary accommodations via a neutral third party. Further, it is important to maintain a high standard of professionalism during job interviews. Departments should ensure that interest is demonstrated in candidates’ research throughout the search process by engaging fully with candidates’ application materials and ensuring that candidates seminars are well attended. Members of the department who interact with candidates should be aware of which questions can and cannot legally be asked during a job interview, including during socialization outside of the formal job interview. Members of the department who interact with candidates should be aware that alcohol can put candidates in an uncomfortable situation, especially because many of the reasons why a candidate may not want to drink relate to the protected identities that are not legal to ask about during job interviews (such as religion and pregnancy), and geoscientists from underrepresented groups are more likely to feel uncomfortable with the amount of alcohol in professional settings [14]. Finally, as many participants were using student interactions to evaluate the department, candidates should have opportunities to interact with students.

Offers and negotiation are an opportunity for candidates to discern how valued they are by the institution. More specifically, institutions can benefit from negotiating in good faith, having a transparent negotiation process, working with candidates’ timelines, finding a strong opportunity for candidates’ partners (if applicable) [15, 16], being polite and respectful toward candidates throughout the process, and offering competitive compensation. More specifically, many participants were looking for enough compensation to buy or rent a home adequate for their family, to be able to afford childcare, to have enough money to travel to see family, and/or to be able to support a partner if a partner hire was not an option. Further, several participants were looking for lab space commensurate with their research goals.

Based on participants’ responses about family, departments and institutions can improve the experience for candidates by being clear about the support systems in place for faculty with partners and children to all candidates, regardless of identity. As mentioned above, helping to secure an exciting opportunity for a partner, if applicable, can help to make an offer more appealing. However, our findings show a mix of outcomes, with many participants declining an offer due largely to a lack of a good opportunity for their partner. This suggests that partner hiring is an important area of improvement for many universities and departments in hiring diverse faculty [15, 16]. Similarly, support for parents has come up in the literature for improving gender diversity [18, 27]. Participants with children expressed additional considerations including sufficient salary and childcare benefits to support children in the university’s location, geographic preferences influenced by raising children, and work-life balance. Some participants mentioned challenges associated with being parents, including low salary offers and being unsure about when to mention their children in a negotiation. This suggests that parenthood is an area of improvement for some universities and departments in hiring. Because many participants were able to get a strong sense of the department culture during their job interviews, and because many candidates are looking for a job with good work-life balance, supporting the work-life balance of existing faculty may be helpful in recruitment.

From the many participant responses about geography, it is clear that geographic preferences played a strong role in the decision to accept or decline an offer for many participants. While an institution cannot easily move to a more desirable location, there may be ways to address candidates’ geographic preferences or concerns, such as through flexible work. Further, because many of the participants’ geographic preferences were tied to politics and personal identities, universities may benefit from working to make their communities desirable places to live for a diverse group of people. How universities may do so (e.g. housing their students and faculty, engaging in politics) is a potentially important area of future research. Preferences of geographic location have come up only briefly in relevant past literature. Oermann et al., 2016 noted the difficulty of hiring nursing faculty in rural locations and Taylor et al., 2010 noted that universities in areas with a high cost of living face challenges recruiting faculty [28, 29]. However, none of this literature is focused on the geosciences specifically or addresses the political considerations that were mentioned by several participants. Therefore, preferences of geographic location and hiring is an area worthy of future study, especially as it relates to political and personal identities.

Departments can improve their hiring process by requesting reference letters late in the process and avoiding really broad searches. Participants generally appreciated when recommendation letters were requested relatively late in the application process. Requesting letters late in the process and reducing their weight may be extra beneficial because women are less likely to receive excellent reference letters than men [30]. Interestingly, several participants were deterred by broad advertisements, which is inconsistent with the notion that broader calls can help diversify the applicant pool [22].

Overall, many of the interventions that have been recommended by previous work, as described in the introduction, were viewed favorably by competitive candidates holding underrepresented racial, ethnic, and gender identities. Therefore, departments are likely to benefit from continued evaluation of hiring practices.

## 4 Methods

### 4.1 Participant recruitment

Our population of interest is geoscientists from underrepresented races, ethnicities and/or genders who declined a tenure-track faculty job at a US institution between 2016 and 2023. To be specific, Black or African American, Asian, American Indian or Alaska Native, Native Hawaiian or Pacific Islander, mixed race, Hispanic or Latino, women, and/or trans or non-binary geoscientists were eligible for our study. Throughout the paper, we use the term “underrepresented” to describe this population of interest, though we recognized that representation and preferred terminology can change over time. Further, we acknowledge that these are not the only identities associated with barriers to successful participation in the geosciences. We interview geoscientists who have declined at least one offer because these geoscientists are both competitive on the job market and have made at least one job decision in their search (i.e., we did not interview geoscientists who selected a job because it was their only option). We interview geoscientists who declined their offer(s) between 2016 and 2023 so that their experiences are relevant to the current job market.

We recruited interview participants using a variety of affinity group and institutional email lists and social media pages. These include the Earth Science Women’s Network (ESWN), the American Geophysical Union (AGU), NSF National Center for Atmospheric Research (NSF NCAR), the Massachusetts Institute of Technology-Woods Hole Oceangraphic Institution (MIT-WHOI) joint program, Asian Americans and Pacific Islanders in Geosciences (AAPIiG), the United States Geological Survey (USGS), and Cryolist. We used this convenience sampling approach because there was no way to develop a complete sampling frame (an exhaustive list of all members of a population to sample from) for our population of interest, as many decisions related to hiring are not made publicly available. To address some of the potential issues with convenience sampling, we used a screening survey (described below) to identify representative participants and ensure balance across our sample. This approach was well-suited for our goal of providing detailed data on a range of hiring experiences. The participant recruitment and interview methods followed standard ethical guidelines and were approved by NSF NCAR’s Human Subjects Committee (HSC). Informed consent was obtained from each participant.

Prospective participants were first asked to fill out a screening survey with basic questions about their job search, their current position, their gender, race, ethnicity, and their willingness to participate in an interview (Appendix A). Based on their responses, survey respondents were invited to participate in a 45 minute interview about their job search if they

- are a geoscientist;
- declined at least one tenure-track faculty job offer between 2016–2023;
- identify as an underrepresented race, ethnicity, and/or gender; and
- were willing to participate in an interview.

This process yielded 19 interview “participants.” We did not interview every white cisgendered woman who met the eligibility requirements because they are overrepresented in our survey responses.

Of the 19 participants, 9 currently hold (or have accepted) a tenure-track faculty position and the other 10 hold a variety of other positions within the geosciences. A variety of disciplines within the geosciences including earth, ocean, atmospheric, and planetary sciences are represented among the 19 participants. Of the 19 participants, 16 identify as an underrepresented gender and 6 identify as a underrepresented race/ethnicity. It is important to note that our sample includes more people with underrepresented gender identities than with underrepresented racial/ethnic identities; White cisgendered women are the most common demographic in our sample. The gender-related and race/ethnicity-related barriers often differ, and combining these aspects of identity into one sample is a limitation of our study. We report results in aggregate to better protect participants’ anonymity.

Participants were free to talk about any experiences they had with hiring, including additional experiences that did not meet the above criteria. Therefore, our findings may include information about experiences with jobs other than tenure-track faculty jobs, jobs outside of the US, hiring experiences before the year 2016, and identities other than race/ethnicity and gender. However, most of the data reported here is about experiences that fit the criteria described above.

### 4.2 Interview methods

Each of the 19 participants participated in an interview of approximately 45 minutes with the lead author of the paper. We used a semi-structured interview protocol to get an overview of the hiring experiences of the participants, while leaving space to probe additional emergent themes [31]. This interview style allows us to draw on a standard list of questions (Appendix B), while allowing the interview to unfold by pursuing concepts raised by participants [32]. Semi-structured interviews are appropriate for this study because they offer the opportunity to hear rich descriptions and detailed information about personal feelings, perceptions, and opinions.

The goal of each individual interview was to determine the ways in which various aspects of the hiring process influenced a participant’s perception of the job opportunity and ultimately why they declined and accepted the offer(s) that they did. To that end, each participant was asked about the logistics of their search, what characteristics they were looking for in deciding to apply for a job, and to summarize the strongest factors that caused them to accept the offer that they did and decline the other(s). Further, each participant was asked more detailed questions about job interview experiences, negotiation experiences, whether and how teaching and DEI came up during application process, any informal contact with the department, about the role of their personal identities in their job search, and any partner, family or caretaking responsibilities that may have influenced their search.

In order to identify main themes, the 19 interviews were recorded and transcribed. We used these recordings and transcripts to examine participants’ strongest reasons for accepting or declining a position, as determined by their response to “briefly, what made you decline the offer(s) that you did and what made you accept the offer that you did?” (Question 6 in Appendix B). Quotes from each response are in Table 10. In answering this question, some participants described what dissuaded them from the offer that they declined, while others described what attracted them to the offer that they accepted, and some described what they were looking for more generally. Many participants described multiple factors in answering this question, and we include them all in Table 10. Some participants either accepted two different jobs at two different times or declined two offers for two different reasons, so quotes outnumber participants in Table 10. Although many participants described factors which are not unique to underrepresented groups, such as resources, teaching load, and hiring process, here we focus only on the themes which are most closely related to personal identities. These themes are: departmental commitment to DEI including personal identities, DEI initiatives, and mentoring; (in)civility during job interviews; values revealed in negotiation; and compatibility with personal life including partner and family and geographic preferences.

After identifying these themes, the lead author made a table that summarized each participant’s responses and included any relevant quotes. Each column of the table corresponds to a one of the themes above. If a participant’s response was relevant to multiple themes, it was included in each relevant column of the table. For each theme the range of responses is summarized in a subsection of Section 2 by both describing the range of responses in the text and highlighting a few exemplary quotes in the tables. Several themes are combined and we do not discuss fit and resources, hiring process, or teaching responsibilities, as these themes may be less influenced by personal identities than the other themes.

For several participants, what they experienced during their job interviews played a pivotal role in their decision (Table 10: Strongest 1–3). One participant declined a job because they were not given enough time to make a decision; the participant was waiting to hear back about other applications (Table 10: Strongest 4). For three participants, a low salary offer dissuaded them from a position (Table 10: Strongest 5–7). Participants’ personal identities and those of the students influenced the decisions of several participants (Table 10: Strongest 8–10). Personal life considerations played a prominent role for many of the participants including a desire to be close to family, a partner’s job, and other geographic preferences (Table 10: Strongest 1, Strongest 5, Strongest 8–18).

In addition, the resources of the department and institution and fit with the colleagues were deciding factors for many participants (Table 10: Strongest 9, Strongest 11, Strongest 13–14, Strongest 17, Strongest 19). Several participants were swayed by the specifics of the faculty job being offered including the ratio of research to teaching (Table 10: Strongest 10, Strongest 16, Strongest 20–21).

In Section 2, we give more detail about participant responses to the themes that relate to personal lives and personal identities: departmental commitment to DEI, (in)civility during job interviews, values revealed during negotiation, and compatibility with personal life. We include responses from participants who did not necessarily identify a theme as one of the strongest factors. However, although they were among the strongest factors for many participants, we do not further discuss departmental and institutional resources or job specifics because they are not as closely tied to personal identities as the other themes.

### 4.3 Limitations

This study describes experiences in the US and focuses primarily on tenure-track faculty jobs. We also focus primarily on experiences between 2016 and 2023. The 2016 to 2023 period included the COVID19 pandemic, which modified the job search process for some participants. Further, the 2016 to 2023 period included the “Me Too” movement and Women’s March in 2017 and the reinvigoration of the “Black Lives Matter” movement following George Floyd’s murder in 2020, which prompted nationwide discussions about diversity and inclusion, especially on college campuses. Therefore, hiring practices may have evolved over this time.

Gender and race/ethnicity are not the only aspects of people’s identities that can be associated with barriers to successful participation. Participants were free to discuss any aspect of their identities, but findings about aspects other than gender and race/ethnicity are not well sampled. Further, we recruited more participants from underrepresented genders (especially cisgendered women) than from underrepresented races/ethnicities. Based on previous work and the findings of this work, the barriers associated with different aspects of identity differ and therefore actions taken to make the geosciences more inclusive to cisgendered women do not necessarily improve inclusivity for other underrepresented groups.

Excluding cisgendered white men from our study comes with limitations. We chose to exclude this demographic because the perspectives of cisgendered white men have historically been well represented in the geosciences. However, cisgendered white men can hold marginalized identities, and geoscientists of all identities can face barriers in the faculty job market. Voluntary participation may have influenced our sample of participants. Further, participants were interviewed by someone in their broad field, and may have adjusted their responses knowing that they may already know their interviewer or with the knowledge that they may encounter the interviewer in the future.

## Figures and Tables

**Table 1:** Quotes from participants about personal identities.

Quote ID	Quote

Identities 1	“I looked very carefully at the demographics of departments I was applying to.”
Identities 2	“Politics and gender and race, for me, have limited where I’m willing to go.”
Identities 3	“It had some very stark lines in terms of where the communities of color were and where the predominantly white communities were. [...] Am I moving my family to a place that will feel safe?” (declined)
Identities 4	“On the grapevine, [they] apparently have a really bad track record with tenuring women and multiple tenured female faculty during my interview unprompted told me how terrible the tenure process had been for them.” (no offer)
Identities 5	“It seems like they were really trying to hire a woman, which is great, but then you’re put in that box.” (declined)
Identities 6	“There was one program in particular became a ‘heck no’ [...] It became a nonstarter. [...] [They] see a checkbox. That’s how it came across. [...] If it’s a numbers game and it’s a checkbox you’re looking for, then am I really truly going to be supported in accepting this opportunity?” (declined)
Identities 7	“I definitely felt tokenized in the sense that I had a meeting with the search committee in which several of the faculty members clearly wanted me to speak about my personal identity. So I ended up coming away not really liking that experience. I thought it was not appropriate.”
Identities 8	“There’s this pioneering woman [in the department where I was interviewing] and I remember thinking about how cool it would be to be her colleague.” (declined)
Identities 9	“The burden of a visa is a horrible stressful burden to carry.”
Identities 10	“They respected me as a queer person.” (accepted)
Identities 11	“My parents don’t have a college degree, so figuring out how to navigate [science] as a career was very challenging.”

**Table 2:** Quotes from participants about DEI in applications and interviews.

Quote ID	Quote

DEI 1	“I really wanted a place that put some effort into diversity.”
DEI 2	“I think the [school where I accepted a job] was the one that was most open to talking about the problem and using the right language, which did affect my feelings about the school. And one of the reasons to choose [to come here], because it seemed like they were genuinely interested.” (accepted)
DEI 3	“I found it surprising that [DEI] was asked about only by the two students that I talked to. The role of the students was to talk about DEI, which felt very odd.” (declined)
DEI 4	“I think the ones that didn’t ask for statements, I’m not sure I got an interview with any of those. And I am pretty active in DEI stuff and even my regular research and teaching statements definitely have DEI stuff in them. It’s curious I didn’t get any interviews with the ones that didn’t require that.”
DEI 5	“I don’t get the feeling that they actually cared or read what I wrote.”

**Table 3:** Quotes from participants about mentorship.

Quote ID	Quote

Mentorship 1	“The most important thing is that [...] both [my Ph.D. and postdoc mentors] believed in me.”
Mentorship 2	“My Ph.D. advisor was exceptionally supportive and I don’t think I would have gotten the jobs without having mentorship from somebody who already has a faculty position who was able to look over my documents and provide feedback.”
Mentorship 3	“In hindsight [being a part of a cluster hire] is a positive because it’s forced me to interact with [...] people outside of my subfield of Earth science, which is great. And it also means that I have a cohort of several other junior faculty.”
Mentorship 4	“They talked about this at the interview, which also led me to want to go there. The first few classes [...] are team taught, so I have mentorship in teaching right away.” (accepted)
Mentorship 5	“Having a mentorship community and having people who care about you coming was way more important than the money to me. As long as you’re at at certain level.”
Mentorship 6	“My postdoc advisor thought because I was a [parent] that I was not gonna be successful in an R1 and refused to help me and told me not to apply to jobs.” (participant is now a professor at an R1 institution)
Mentorship 7	“They’re looking for someone to coordinate [one of their degree] programs [...] and I got really excited about that aspect of it.” (declined)
Mentorship 8	“I was hoping to work in a place where the institution and my colleagues cared about teaching and mentoring well.”

**Table 4:** Quotes from participants about job interview experiences.

Quote ID	Quote

Interviews 1	“Someone not on the hiring committee reached out from a DEI perspective before I went to the on-campus interview and they [asked] ‘are there any accommodations that you need?’ That was, both new and very positive [for] understanding that department culture.” (declined)
Interviews 2	“We had casual time and dinner with people not on the committee who were very friendly and open, but no boundaries seemed to be crossed.” (accepted)
Interviews 3	“[I had] a mixed experience meeting the different faculty. Some faculty just didn’t show up for anything, some faculty were there the whole time and I spent a lot of time with them.” (declined)
Interviews 4	“[There] was the lack of camaraderie that I had been able to glean from any of the faculty, even sitting around a dinner table sharing a meal together.” (declined)
Interviews 5	“I’ve been really attracted by some departments that clearly are very cohesive and work together closely and put off by some departments that seem to have a real dichotomy.”
Interviews 6	“I was particularly paying attention to interactions with students during my interviews.”
Interviews 7	It was a “red flag” that “there were no students involved [in the interview].” (accepted and has since left)
Interviews 8	“For interviewers: use real students [during teaching demos], it works better.”
Interviews 9	“I ended up second choice for the job largely because I questioned the department chair about [a professor in the department with a reputation for inappropriate behavior]. [...] I have some friends there [and] that seems like that was potentially a make or break on if I got the job or not.” (no offer)
Interviews 10	“During one of the interviews I was asked my sexuality, my religion, if I was currently pregnant, and maybe if I was married. I like to believe that they were asking with good intentions, [...] but I was appalled.” (no offer)
Interviews 11	“The worst one was when I was at an interview, we went to [a meal and] I was one on one with an older professor who told me that the only reason I’ve made it so far in my career was how I looked. And made some not appropriate comments about being a [person of my identity] in science.” (declined)
Interviews 12	“We were talking about courses that I could teach and [someone from the department] basically said, ‘well, you’re not [this type of scientist] so you wouldn’t be able to teach any courses [on that subject]’. But I’m like, ‘well, that’s what I do.’”
Interviews 13	During an interview, one professor “basically insinuated that I was lying about the [DEI work] that I did.” (declined)
Interviews 14	“People forgot to show up for my scheduled times. People were late picking me up. People were late to dropping me off at the next thing. I had no control over any of it.” (no offer)
Interviews 15	“I hardly came across people who seemed like they had read any of the statements I had submitted.”
Interviews 16	“Multiple senior faculty just no-showed their meetings with me. Like I went to their door and they weren’t there.”
Interviews 17	“I did have a drink at every dinner [even though I did not want to because of a personal identity]; it was definitely a pressure that I was not happy to have.”

**Table 5:** Quotes from participants about negotiation.

Quote ID	Quote

Negotiation 1	“It was all a little awkward with [the university I was negotiating with] in the sense that they don’t make [me] an offer to start with. They basically want[ed me] to say what I needed to do what I said that I would do. And so there was all of this interpretation exercise of trying to figure out what I should [request] for startup.” (declined)
Negotiation 2	“One thing I wish that’d been better in the negotiation process for parents is, well, I didn’t know when to say I was a [parent]. [...] It turns out I could have negotiated childcare. [...]. I didn’t want to say anything until an offer letter was signed, but then I missed out on being able to get [it].” (accepted)
Negotiation 3	“Specifically about two bodies: in some situations I’ve spoken with faculty and they’re like ‘I just wish people would tell us ahead of time if they have an accommodation need because it helps us provide them a better offer, which we can’t do if we don’t know.’ And then other people have been like ‘Yeah, I don’t tell them because I’ve actually told them in the negotiation that I had a spousal accommodation and the job offer disappeared.’ So there’s so many different ways in which it plays out.”
Negotiation 4	“A lot of it came down to the specifics of the offer that they did give my partner. It wasn’t really like what [my partner has] here, so that was a big factor.” (declined)
Negotiation 5	“They were literally losing a faculty member because of a two-body problem and they were unwilling to talk to me about how to accommodate a two-body problem beyond a few condescending suggestions.” (declined)
Negotiation 6	“What was crazy, there was one institution where I had a friend there and I was warned that [people of my identity] coming in had been lowballed. And I thought the salary was low. I asked for [a very large increase in salary] and they said yes, without even thinking about it. That played in my role of making that decision. They weren’t even giving a fair market rate.” (declined)
Negotiation 7	“[I] knew I had an offer, but they were very brusque about it. Like, ‘we’re fine if you don’t come here to just, we don’t want to waste time’. It was not far off from those words. So [I thought] ‘well, I’m not sure if I would feel valued’.” (declined)
Negotiation 8	“I had asked for a course release, but [they] said something like ‘Oh, I’m not sure about that. I mean, if that’s really important to you, I’d be happy to bring that up at the next faculty meeting, but we may need to rescind your offer’.” (accepted)
Negotiation 9	“There was a more senior faculty member who made quite disparaging comments about my ability to start a lab, which made it just really easy to say no to that place.” (declined)
Negotiation 10	“The initial offer was so low that it wasn’t worth negotiating.” (declined)
Negotiation 11	“When the people offering you the money make four times as much and don’t see why that should matter [...it] suggests to me that it will show up in other ways.” (declined)
Negotiation 12	“It was less than I was making as a postdoc.” (declined)
Negotiation 13	“A [prestigious private R1] institution cannot solve basic problems. If this is the best that a [prestigious private R1 institution] has to offer, maybe we should think about it a little harder.” (declined)
Negotiation 14	“When I got the offer there was no opportunity to negotiate. They basically handed me an offer that included a salary and the startup and I had to decide to accept or decline it in two weeks. I don’t think that’s super common and I wasn’t expecting that.” (declined)
Negotiation 15	“The timing matters.”

**Table 6:** Quotes from participants about family.

Quote ID	Quote

Family 1	“I think that there are difficulties that come with being single in a new environment just as there are difficulties when trying to move as a couple or trying to move with kids.”
Family 2	“My [partner] gets a vote.”
Family 3	“I wouldn’t have taken any of these jobs if there hadn’t been an offer for my [partner].”
Family 4	“I was advised by older faculty [. . . not to] mention that I was married at all. I didn’t mention that I had children at all. [...] I just kept my personal life very out of it. Nobody knew I had children, which made it a little easier, but it influenced my decision making. [...] I wish I could be more honest in the interviews, but I know you’re not supposed to.”
Family 5	“One of my meetings was with professors who had children and they said, ‘We’re not asking you anything, but here we’re just gonna tell you about our experiences with tenure clock extension and everything.’ And that was really helpful.” (accepted)
Family 6	“Being in a large city where it’s easier to meet people, where there are more people, and then having family nearby, that network is sort of built-in. [...] All of that really helps alleviate some of the loneliness that comes with not being in a [...] relationship.” (accepted)
Family 7	“Finding a place that I felt aligned with the work-life balance I envisioned, I think that was really important. [...] I think seeing other people at dinner talk about their kids or their hobbies or how they balance their work-life like it was a very open topic. I think that was always very encouraging [and that it] showed that it was a topic which people were thinking about.”

**Table 7:** Quotes from participants about geographic preferences.

Quote ID	Quote

Geography 1	“I felt that being geographically picky was not a luxury that I had.”
Geography 2	“I almost feel bad for even thinking about location.” (declined)
Geography 3	“I think the overarching state politics gave me pause at a couple of the places.”
Geography 4	“Will I be at a school where my hands are tied in terms of how I teach a course like climate change?”
Geography 5	“I valued feeling safe in the community. And I think that was lacking in a couple of the places [and] that push[ed] that onto the ‘no’ list for me.”
Geography 6	“In terms of what’s occurred recently, [the city where the university is located] has been one of the unfortunate many cities in the racial spotlight.” (declined)
Geography 7	“It was a little bit hard to imagine living in a place that [remote and not diverse] with a baby for a really long time.” (accepted then left for a different job)

**Table 8:** Quotes from participants about other themes.

Quote ID	Quote

Other 1	[I avoided really broad advertisements and limited the number of places I applied to because] “it’s a waste of time to submit an untailored application.”
Other 2	Broad calls are “just for them to go fishing and see what they can catch.” (applied to broad calls but felt it was a waste of time)
Other 3	“I had some hesitation about applying because [...] I didn’t want to put undue load on my reference writers at the application stage. That dissuaded me at some places. [...] I appreciate [...] that more and more they would only contact the referees right before [...] the in-person interview stage.”
Other 4	“I also avoided some departments where I knew there were real a**holes that were faculty.”
Other 5	“There are places I will not even consider because they don’t make consequences happen to faculty who are behaving unacceptably.”
Other 6	“Hearing about [a friend’s] overall very positive experience made me more excited about the position.”
Other 7	“In academia, job searches can get very personal. [...] Accepting them into their department or if someone leaves it’s very dramatic or declining is a big deal. [...] It can hamper professionalism, I think, because how personally people take it (on both sides).”
Other 8	“There have been some tragic incidents where students have acted aggressively toward faculty.”
Other 9	“Both during the job search and especially once I got this job I experienced a pretty significant amount of imposter syndrome. Especially because this was the job that was really the dream one that I wanted the most and a lot of other people applied to it. It’s almost impossible to not question why you got it.”
Other 10	“There are going to be all these people who think I’m crazy for turning down a tenure-track faculty position.”

**Table 9:** Summary of recommendations.

**Respect personal identities**
• Avoid tokenizing candidates
• Be aware of invisible identities
• Use correct pronouns
• Support international faculty in securing a visa (if applicable)

**Support departmental DEI efforts**
• Diversify the department at all levels
• Be well-informed about DEI issues
• Encourage senior faculty to participate in DEI efforts

**Improve and communicate mentorship programs**
• Mentor junior faculty, including in teaching
• Encourage and support faculty in mentoring students and postdocs
• Offer mentorship in teaching for new faculty

**Improve underlying departmental issues**
• Improve student satisfaction
• Improve faculty satisfaction
• Improve work-life balance
• Improve department cohesion
• Reduce unprofessional behavior
• Eliminate misconduct

**Increase departmental awareness of hiring best practices**
• Avoid asking illegal questions
• Avoid disparaging behavior toward candidates
• Offer candidates accommodations via a neutral party
• Maintain professionalism during interviews
• Engage fully with candidates
• Avoid alcohol

**Negotiate in good faith**
• Make the negotiation process transparent
• Work with candidates’ timelines and individual preferences
• Accommodate the desires of candidates’ partners (if applicable)
• Be polite and respectful throughout the negotiation
• Offer competitive compensation
• Give candidates sufficient time to make a decision

**Improve and communicate support for partners and children**
• Facilitate finding an exciting employment opportunities for partners (if applicable)
• Improve and communicate support for parents
• Improve and communicate support for work-life balance

**Make the hiring process candidate friendly**
• Request letters of recommendation for finalists only
• Avoid broad searches

**Table 10:** Quotes from participants about the strongest factors influencing their decision to accept or decline an offer.

Quote ID	Quote

Strongest 1	“A huge one was geography. It was one of my only offers that was in [a region of the US which was desirable to me]. During my interview people were very personable, genuinely interested in my research, generally had read my things, [and mentioned] providing resources for support for grants. [...] In my interview at [my current institution], I was not asked any inappropriate questions. There was no mention of my [identity], there were no problems of those sort, which is not true at nearly all the other schools I interviewed at.”
Strongest 2	“I didn’t get a good vibe. It was a very large college so I felt that it would be hard to thrive. It was just like one cog in a very large machinery.” (declined)
Strongest 3	“I went and I did the interview and I just had a really bad, awkward feeling from the interview. [...] A bunch of people were away and so I didn’t really get to meet a lot of people.” (declined)
Strongest 4	“Even though it was quite highly-ranked in [a] place that I wanted to go, it just expired.” (declined)
Strongest 5	“It was a pay cut and a move and there really wasn’t a negotiation. The biggest thing my partner and I decided on was that [my partner] really needed the opportunity to be able to relocate to a place that would support [them]. [...] Le’s see what they say about making an accommodation for [my partner]. [...] The response was really underwhelming.” (declined)
Strongest 6	“Living in [that location] on the salary that they were offering was just not a viable option.” (declined)
Strongest 7	“Money. Both offers offered me less than I was currently making per year as a postdoc.” (declined)
Strongest 8	“Two main reasons. One was location; one [job] was closer to family. And the other main reason was the student population. My current institution has the most diverse student population I’ve ever encountered and I really wanted to be in an institution that valued that.” (accepted)
Strongest 9	“The timing was a factor. [...] A job for my partner was a huge consideration. Then I started to really think about location, whether it would be closer to family, whether we wanted to live in that place. Of course I got more information when I visited in person. [...] [at the jobs that I declined] there might not be too many people that do what I do. And the demographics of the different departments. One was very male dominated, the other [was] more mixed. [I was] thinking about the overall environment, colleagues, the job duties, things like teaching loads, there are so many factors.”
Strongest 10	“Definitely the teaching component was one that I was less interested in. [...] I did have some pause and concern about ‘How safe are college campuses in this country in this day and age?’. Coupled with the racial, political side of the equation as well. [...] Geography certainly weighed on it as well. [...] It was definitely one of the tougher ones to turn down because it is a prestigious institution.” (declined)
Strongest 11	“It was the a combination of the geography [being near my partner’s family] and then the prestige and the quality of students and of colleagues that I would have that really made it a no brainer.” (accepted)
Strongest 12	“The location. My [partner] wanted to move to [this location]. I mean, [my partner’s] entire family on both sides [lives nearby].” (accepted)
Strongest 13	“Resources and geography. I think both departments have great department culture. They both wanted someone of my flavor of [research]. And both would have been great institutions to to join. [...] It’s resources like the ability to pay students and hire postdocs and really get my lab ramped up. [...] Being on one of the coasts was somewhat important [to my partner].”
Strongest 14	“It was just the sense of this really awesome community and all these intellectual opportunities because there’s so many people thinking about related science from different directions. That was the most exciting professionally. And then personally this is a great fit for what I was looking for from a geography perspective.” (accepted)
Strongest 15	“The department had not hired anyone at the assistant professor level in [many] years. [It] made me hesitate and question about the sorts of things were happening. And then I have a partner who [has a career]. And we soon realized that there weren’t that many options [for my partner in that city].” (declined)
Strongest 16	“I often think that one of the hardest things I’ve ever done was turn down the [tenure-track faculty job] offer, just because I was like ‘This could be my only opportunity to be a professor’. But I think I ultimately realized that I’d rather not be a professor than have a [large] course load, expected to teach classes that I didn’t feel comfortable with and live in [the city where the job would have been located].” (declined)
Strongest 17	“It became a matter of ‘can [my partner and I] both have jobs in this place?’. And then the next step is [whether or not] it has an intellectual environment that is really meaningful to me.”
Strongest 18	“I was given [and accepted] a retention offer which was better only in that it didn’t involve me having to move across country and then be further away from my partner.” (accepted)
Strongest 19	“The university [where I declined an offer] is little less well resourced, they didn’t have the same kinds of resources for research and they weren’t able to draw the same kinds of graduate student applicants that [university where I accepted an offer] did. Also, I actually really enjoy being in a big department such as [this one]. [...] I think it’s been fun for me and for my graduate students to have that sort of community and critical mass people.”
Strongest 20	“I got an offer from a SLAC [small liberal arts college] [and an R1]. And the main reason why I ended up going with the [R1] is I realized after really talking to faculty at SLACs that I did want a job that was more research focused.”
Strongest 21	“The biggest draw to me here is that teaching is equally [as] valued [as the] research aspect. [We’re] encouraged to continually improve [our] teaching and think about that deeply as opposed to a lot of, say, R1 schools where your focus is research and you have to teach as one of those obligations.” (accepted)

## Data Availability

Given the confidential nature of this work, the data cannot be made available.

## Footnotes

1 This includes people who identify as Black, Hispanic, Native American, and/or Asian

2 In our terminology, we use ”department” to describe the entity which is conducting the search and interviews and ”institution” to describe the entity which is doing the negotiation.

3 Tokenism is the policy or practice of making only a symbolic effort.

## Appendix A Survey questions

- Which best describes your area of research? (Earth Science, Ocean Science, Atmospheric Science, Planetary Science, Other)
- What is your current position?
- How many tenure-track faculty offers in the geosciences have you declined (If possible, within the last 7 years)?
- Asked for each declined offer: In the spaces below, please input the name of a university and department from whom you declined an offer, as well as the year that the offer was made to you.
- What is your gender?
- Are you Hispanic or Latino (yes, no)?
- What is your racial background? (Participants could select as many options as they like from the following list: White, Black or African American, American Indian or Alaska Native, Asian, Native Hawaiian or Pacific Islander, Other)
- Would you be willing to participate in a 45 minute virtual interview about your experience applying for a job in the geosciences?
- If so, please enter your name and email address below.

## Appendix B Interview questions

1. Before we start talking about the search for a permanent job, tell me a little bit about your journey into the geosciences. (This question was intended as a warm up.)
2. Where were you when you started applying for your current position?
3. How many jobs did you apply for and how many of them were faculty jobs?
4. Were you sure you wanted a tenure-track faculty job at the time that you applied for your position? Were you considering other kinds of positions?
5. What characteristics were you looking for when deciding to apply or not to apply for a job? This can include characteristics that pertain to your personal life.
6. Briefly, what made you decline the offer(s) that you did and what made you accept the offer that you did?
7. Were there any aspects of the interview process that made you more or less interested in a job? This can include interviews for jobs other than your current position.
8. Were there any aspects of the offer or negotiation process that made you more or less interested in a job? This can include offers and negotiations for faculty jobs other than your current position.
9. Did you have any other contact with academic departments that were hiring that influenced your decision (e.g. Invitation to apply, Conversations with members of that department)?
10. Were you asked to talk about DEI in your application materials or interviews?
11. Were you asked to talk about teaching in your application materials or interviews?
12. How do you think your personal identities influenced your faculty search process? Your answer does not have to be limited to race and gender.
13. Did a partner, family, or caretaking responsibility influence your job search?
14. Is there anything else you would like to share with us about your faculty job search?
